# Baihe Dihuang Tang Exerts Antidepressant Effects via Modulation of MAOA-Mediated Serotonin Metabolism and Synaptic Plasticity

**DOI:** 10.3390/ph18121786

**Published:** 2025-11-24

**Authors:** Defu Tie, Yuting Wang, Jieru Zhou, Yiting Zhang, Hua Ji, Yue Yu, Haijun Han, Zheng Xiang, Wenlong Li

**Affiliations:** 1 College of Pharmaceutical Engineering of Traditional Chinese Medicine, Tianjin University of Traditional Chinese Medicine, Tianjin 301617, China; defutie1122@163.com; 2 School of Medicine, Hangzhou City University, Hangzhou 310015, China

**Keywords:** depressive disorder, Baihe Dihuang Tang, network pharmacology, 5-HT metabolism, synaptic plasticity

## Abstract

**Background/Objectives:** Baihe Dihuang Tang (BDT), a classical herbal formula from Zhang Zhongjing’s Han Dynasty work *Jin Gui Yao Lue*, is widely used to treat depressive disorder by nourishing Yin, clearing heat, and tonifying the heart and lungs. However, its pharmacological mechanisms remain unclear. This study aims to explore BDT’s antidepressant effects via MAOA-regulated serotonin (5-HT) metabolism and synaptic plasticity, supported by experimental validation, while using network pharmacology to predict MAOA-targeting active components. **Methods:** Active components and targets of BDT were screened using TCMSP, TCMID, and other databases, and then a component-target-pathway network was constructed. A chronic restraint stress (CRS)-induced depressive mouse model was established. Behavioral tests, including open field test (OFT), elevated plus maze (EPM), forced swimming test (FST) and tail suspension test (TST), were conducted to evaluate antidepressant effects. ELISA, qRT-PCR, and Western blot were employed to assess hippocampal 5-HT metabolism (MAOA, 5-HT/5-HIAA ratio) neurotrophic signaling (BDNF, TrkB) and synaptic plasticity-related proteins (PSD-95, SYN1). **Results:** BDT significantly reduced FST/TST immobility time and improved anxiety-like behaviors in OFT/EPM. BDT treatment downregulated MAOA expression, elevated hippocampal 5-HT/5-HIAA ratio, activated BDNF/TrkB pathway, and upregulated PSD-95/SYN1. Network pharmacology confirmed MAOA’s central role, identifying MAOA/serotonergic synapse modulation as BDT’s main mechanism and pinpointing Ferulic acid, Caffeate, Stigmasterol, (−)-nopinene, Eugenol, and cis-Anethol as MAOA-targeting bioactive components. **Conclusions:** BDT ameliorates depressive-like behaviors. This effect is mechanistically linked to suppression of MAOA-mediated 5-HT catabolism—a key validated target. This suppression elevates hippocampal 5-HT bioavailability, thereby activating BDNF/TrkB signaling and promoting synaptic plasticity. Network pharmacology confirmed MAOA as a primary target and identified specific modulatory bioactive components.

## 1. Introduction

Depressive disorder represents a major global public health challenge. The World Health Organization (WHO) epidemiological data reveal that over 350 million individuals are affected by depressive disorder globally, with depressive-associated suicide claiming approximately 800,000 lives annually. Alarmingly, the global age-standardized incidence rate of depressive disorder has increased by 12.9% from 1990 to 2019, while the average age of onset has shifted toward adolescence and early adulthood [1]. One in five people (22%) who have experienced war or conflict in the previous 10 years has depression, anxiety, post-traumatic stress disorder, bipolar disorder or schizophrenia [2]. Although selective serotonin reuptake inhibitors (SSRIs) remain first-line pharmacotherapies, their clinical utility is constrained by a 2–4-week latency in therapeutic onset and adverse effects (e.g., sexual dysfunction, weight gain) that lead to 30–50% discontinuation rates. necessitating the development of multitarget agents with rapid antidepressant effects and favorable safety profiles [3].

Traditional Chinese medicine (TCM) offers distinct therapeutic benefits in treating mild-to-moderate depressive disorder due to its safety and holistic regulatory effects. Classic herbal formulae including Baihe Dihuang Tang (BDT) [4,5] and Xiaoyaosan [6,7] are widely used in clinical practice for depressive disorder management. BDT was first documented in the Han Dynasty medical treatise *Jin Gui Yao Lue* (*Synopsis of the Golden Chamber*) by Zhang Zhongjing, consisting of two pharmacopeial herbs: *Lilium brownii* var. *viridulum* (Baihe, lily bulb) and *Rehmannia glutinosa* (Sheng Dihuang, raw rehmannia root), both listed in the Chinese Pharmacopoeia for their dual medicinal and dietary applications. Recognized for its dual functions of nourishing yin, clearing heat, and tonifying the heart-lung system, BDT stands as one of the most frequently prescribed formulas for depressive disorder in TCM practice [8,9]. Clinically, it has shown efficacy in alleviating depressive symptoms, insomnia, anxiety, and menopausal syndrome-related psychiatric disorders [10].

The monoamine hypothesis proposes that functional deficits in monoaminergic neurotransmitters, such as serotonin (5-hydroxytryptamine, 5-HT) and norepinephrine (NE), underlie a central pathological mechanism of depressive disorder [11]. Studies indicate that monoamine oxidase A (MAOA), which catalyzes 5-HT degradation (to 5-hydroxyindoleacetic acid, 5-HIAA) and reduces synaptic neurotransmitter availability, represents a crucial therapeutic target for antidepressants [12]. Existing studies have demonstrated therapeutic efficacy of BDT in alleviating depressive symptoms through diverse mechanistic pathways. For instance, Feng et al. demonstrated that BDT ameliorates depressive-like behaviors in rats by regulating amino acid, steroid hormone balance, and glycerophospholipid homeostasis [13]. Further evidence indicates that BDT attenuates anxiety- and depressive-like behaviors in chronic unpredictable stress (CUS) mice via AMPA receptor activation [14], while its modulation of miRNA-144-3p-mediated GABAergic transmission effectively reverses somatostatin (SST)-positive neuronal deficits [4]. Additionally, BDT corrects gut microbiota dysbiosis and suppresses inflammatory responses linked to intestinal barrier dysfunction, thereby ameliorating depressive-like behaviors and synaptic impairments [15]. However, despite these advances, the specific effects of BDT on monoaminergic neurotransmission, a cornerstone of depressive pathology, remain underexplored. Notably, comprehensive understanding of its dynamic regulation of monoamine synthesis, metabolic clearance, and downstream signaling transduction awaits further mechanistic elucidation.

This study aimed to elucidate the antidepressant mechanisms of BDT by investigating its regulatory effects on 5-HT catabolism and synaptic plasticity. Using a chronic restraint stress (CRS) mouse model integrated with network pharmacology and experimental validation. The findings provide mechanistic evidence supporting therapeutic efficacy of BDT in depression and offer a foundation for optimizing its clinical application through targeted modulation of 5-HT metabolism and synaptic function.

## 2. Results

### 2.1. Results of Quality Control of BDT

**Figure 1:**
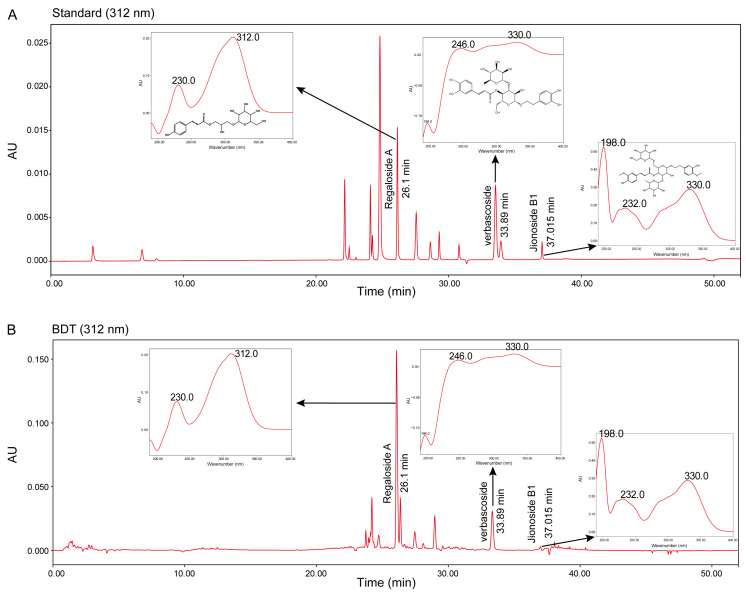
Determination of jionoside B1, regaloside A, and verbascoside in BDT. (**A**) Retention times (from left to right) and UV absorption peaks of jionoside B1, regaloside A, and verbascoside in a mixed standard. (**B**) BDT chromatogram (312 nm) showing UV absorption peaks corresponding to jionoside B1, regaloside A, and verbascoside.

To ensure the stability and controllability of BDT, this study utilized high-performance liquid chromatography (HPLC) to analyze its chromatographic profile, using reference standards including jionoside B1 (from *Rehmannia glutinosa*), regaloside A and verbascoside (from *Lilium brownii*). As shown in Figure 1A, the retention times and maximum absorption wavelengths of the reference standards were as follows: regaloside A at 26.1 min (312 nm), jionoside B1 at 37.051 min (198 nm), and verbascoside at 33.89 min (330 nm). Under identical conditions, the retention times and absorption peaks of these three compounds in BDT (Figure 1B) matched those of the reference standards, confirming the presence of these components in BDT and ensuring the quality of the herbal formulation.

### 2.2. Results of Behavioral Testing

**Figure 2:**
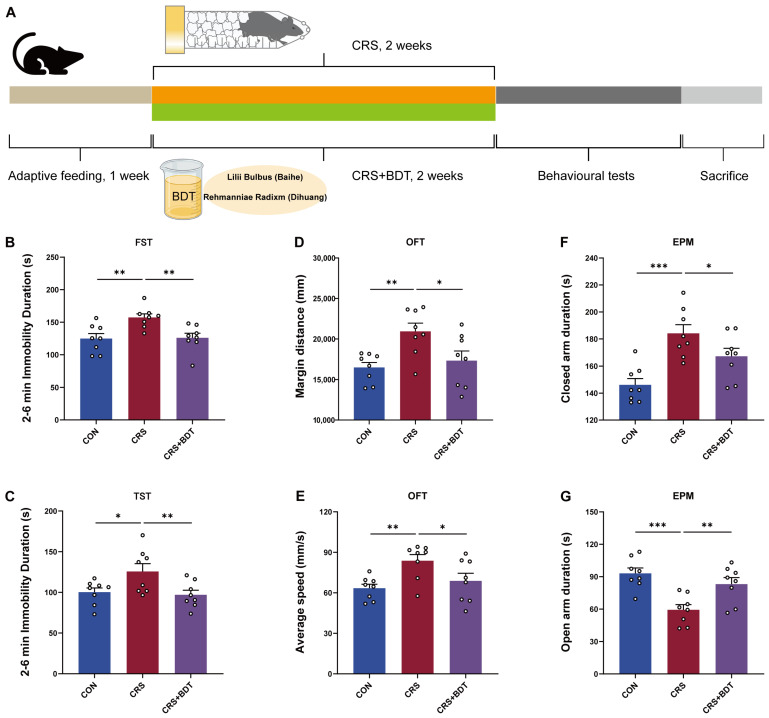
Animal experiments and behavioral assessments. (**A**) Schematic diagram of the CRS modeling and drug administration protocol (Beige: 1 week adaptive feeding, Orange: 2 weeks CRS modeling, Green: 2 weeks concurrent BDT treatment, Dark gray: behavioral assessments, Light gray: tissue harvesting). (**B**) FST: Mice were subjected to a 6 min test, and immobility time (indicating behavioral despair) during the last 4 min was analyzed. (**C**) TST: Immobility time was measured using a protocol similar to FST. (**D**,**E**) OFT for anxiety-like behaviors. (**D**) Total distance traveled in the peripheral zone. (**E**) Average speed of mice in the open field. (**F**,**G**) EPM for anxiety-like behaviors. (**F**) Time spent in closed arms. (**G**) Time spent in open arms. * *p* < 0.05, ** *p* < 0.01, *** *p* < 0.001.

To evaluate the effects of BDT on depressive- and anxiety-like behaviors, the forced swim test (FST) and tail suspension test (TST) were used to assess behavioral despair in mice. Compared with the control group, mice exposed to chronic restraint stress (CRS) showed significantly increased immobility time in both the FST (*p* < 0.01) and TST (*p* < 0.05). BDT treatment significantly reduced immobility time in CRS-exposed mice (*p* < 0.01 vs. CRS group in both tests), demonstrating antidepressant-like efficacy (Figure 2B,C).

Anxiety-like behavior was further assessed using the open field test (OFT) and elevated plus maze (EPM). In the OFT, CRS mice exhibited increased path length in the peripheral zone (*p* < 0.01) and elevated average speed (*p* < 0.01) compared to controls. Both parameters were significantly normalized by BDT treatment (*p* < 0.05 vs. CRS group; Figure 2D,E). In the EPM, CRS mice spent more time in the closed arms (*p* < 0.001) and less time in the open arms (*p* < 0.001) relative to controls. BDT administration increased open-arm time (*p* < 0.01) and reduced closed-arm time (*p* < 0.05) compared to the CRS group (Figure 2F,G). These results indicate that CRS induces both depressive- and anxiety-like behaviors in mice, and that BDT effectively counteracts these deficits, supporting its potential dual therapeutic benefit for mood disorders.

### 2.3. BDT-Mediated Effects on MAOA

**Figure 3:**
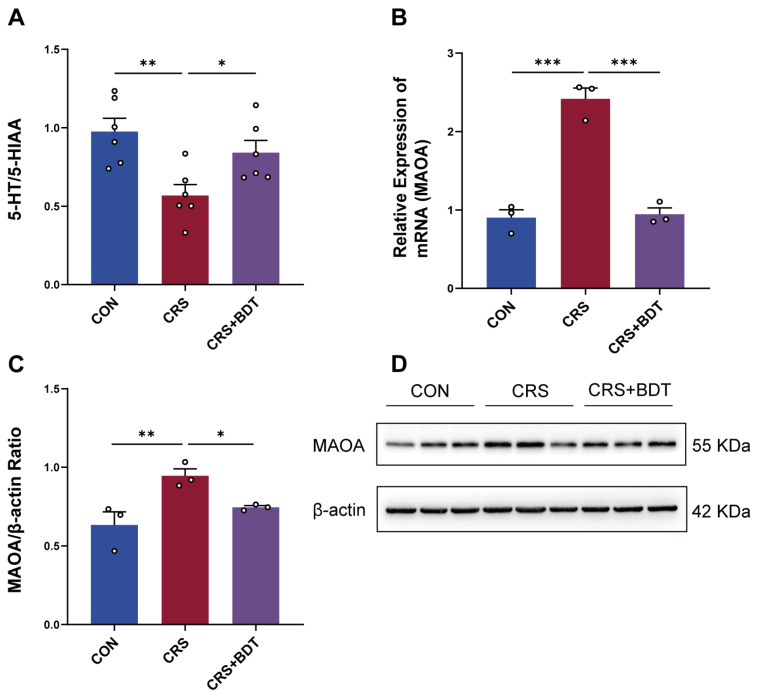
Effects of BDT on MAOA and 5-HT metabolism. (**A**) Ratio of 5-HT/5-HIAA in the hippocampal tissue of mice, reflecting 5-HT metabolic activity. (**B**) MAOA mRNA expression levels in the hippocampus measured by qRT-PCR. (**C**,**D**) MAOA protein expression levels in the hippocampus analyzed by Western blot. Representative blots (**C**) and quantitative data (**D**) are shown. * *p* < 0.05, ** *p* < 0.01, *** *p* < 0.001.

To elucidate the mechanisms underlying BDT’s modulation of serotonergic synapses, serotonin (5-HT) and its metabolite 5-hydroxyindoleacetic acid (5-HIAA) were quantified in hippocampal tissue using ELISA. The calculated 5-HT/5-HIAA ratio was significantly reduced in CRS mice compared with controls (*p* < 0.01), indicating impaired serotonin metabolism. BDT treatment restored this ratio (*p* < 0.05 vs. CRS group), suggesting enhanced serotonin bioavailability (Figure 3A).

To investigate the molecular basis of this effect, the expression of monoamine oxidase A (MAOA), a key enzyme in serotonin degradation, was analyzed. Quantitative reverse transcription polymerase chain reaction (qRT-PCR) revealed that MAOA mRNA levels were upregulated in CRS mice (*p* < 0.001 vs. control group), an effect that was downregulated by BDT treatment (*p* < 0.001 vs. CRS group; Figure 3B). Western blot analysis confirmed corresponding changes at the protein level: MAOA expression was elevated in the CRS group (*p* < 0.01 vs. controls) and significantly reduced by BDT administration (*p* < 0.05 vs. CRS group; Figure 3C,D).

These results suggest that BDT alleviates depression-like behaviors by inhibiting MAOA-mediated serotonin degradation, thereby increasing synaptic serotonin availability.

### 2.4. Impact of Serotonergic Synapse on BDNF/TrkB

**Figure 4:**
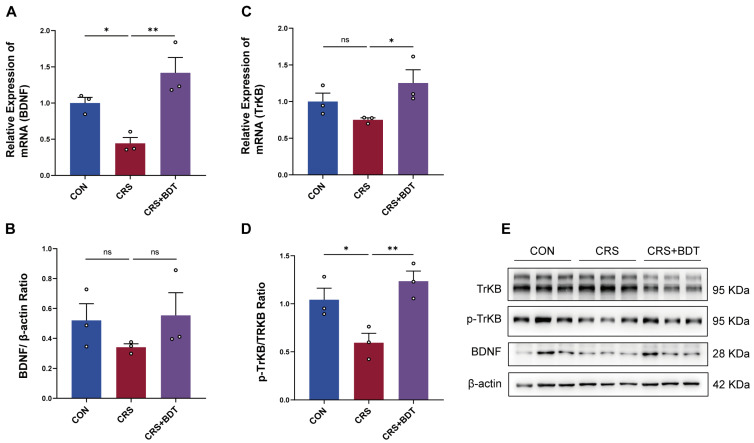
Effects of BDT on neurotrophic factors. (**A**) BDNF mRNA expression levels in the hippocampal tissue measured by qRT-PCR. (**B**) BDNF protein expression levels in the hippocampal tissue analyzed by Western blot. (**C**) TrkB mRNA expression levels in the hippocampal tissue assessed by qRT-PCR. (**D**) TrkB protein expression levels in the hippocampal tissue determined by Western blot. (**E**) Blots depict TrKB, p-TrKB, BDNF, and β-actin across CON, CRS, and CRS+BDT groups. Molecular mass markers denote 95 kDa for TrKB and p-TrKB, 28 kDa for BDNF, and 42 kDa for β-actin. * *p* < 0.05, ** *p* < 0.01, ns: non-significant differences.

To investigate the neuroprotective mechanisms of BDT, we focused on key nodes of serotonin signaling. Experimental data revealed that 5-HT activates downstream pathways by binding to the HTR2B receptor, driving brain-derived neurotrophic factor (BDNF) expression and promoting its receptor (neurotrophic receptor tyrosine kinase 2, TrKB) phosphorylation (p-TrkB) [16], a process critical for neuronal survival and functional maintenance. In CRS mice, hippocampal BDNF mRNA levels were significantly downregulated (*p* < 0.05 vs. control), while TrkB mRNA levels remained unchanged (Figure 4A,C). Strikingly, BDT intervention upregulated both BDNF (*p* < 0.01 vs. CRS) and TrkB (*p* < 0.05 vs. CRS) mRNA expression, suggesting dual regulatory effects on neuroprotective genes.

At the protein level, BDNF exhibited a trend of upregulation consistent with mRNA changes but did not reach statistical significance. p-TrkB protein levels were reduced in CRS mice (*p* < 0.05 vs. control), and BDT treatment significantly reversed this decrease (*p* < 0.01 vs. CRS group) (Figure 4D,E). This dissociation between transcriptional and post-translational regulation implies that BDT not only enhances BDNF/TrkB signaling at the transcriptional level but also restores impaired TrkB pathway activity through post-translational modifications, such as phosphorylation. The antidepressant effects of BDT are mediated through modulation of the 5-HT-dependent BDNF-TrkB neuroprotective axis, which restores neurotrophic homeostasis and optimizes receptor signaling dynamics. These findings demonstrate that BDT exerts its antidepressant and neuroprotective effects by multi-level potentiation of the 5-HT-BDNF-TrkB axis, effectively restoring neurotrophic homeostasis through coordinated transcriptional and post-translational regulation.

### 2.5. BDT-Mediated Effects on Synaptic Plasticity

**Figure 5:**
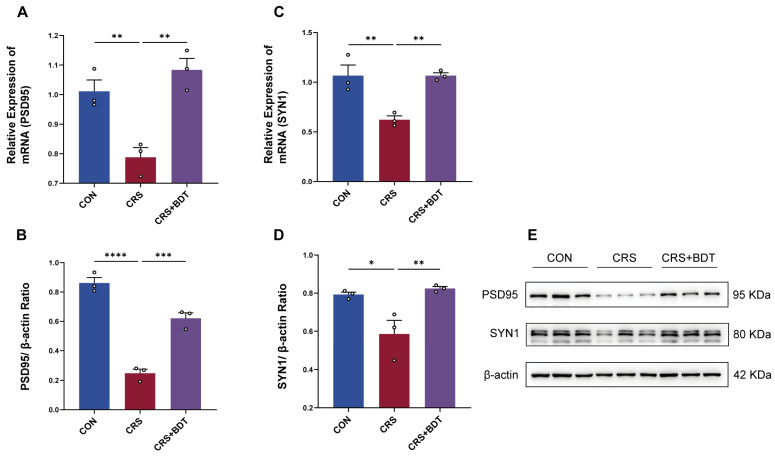
Effects of BDT on synaptic plasticity. (**A**) PSD-95 mRNA expression levels in the hippocampal tissue measured by qRT-PCR. (**B**) PSD-95 protein expression levels in the hippocampal tissue analyzed by Western blot. (**C**) SYN1 mRNA expression levels in the hippocampal tissue assessed by qRT-PCR. (**D**) SYN1 protein expression levels in the hippocampal tissue determined by Western blot. (**E**) Blots depict protein levels in CON, CRS, and CRS+BDT groups. Molecular masses are annotated as 95 kDa for PSD95, 80 kDa for SYN1, and 42 kDa for β-actin. * *p* < 0.05, ** *p* < 0.01, *** *p* < 0.001, **** *p* < 0.0001.

BDT activates the BDNF/TrkB signaling pathway, triggering a cascade of downstream signaling events critical for synaptic plasticity regulation. This mechanism is closely associated with the expression modulation of key synaptic proteins, including postsynaptic density protein 95 (PSD-95) and synapsin 1 (SYN1) [17]. In the CRS model, hippocampal PSD-95 and SYN1 mRNA levels were significantly downregulated (both *p* < 0.01 vs. control), whereas BDT intervention restored their expression (both *p* < 0.01 vs. CRS group) (Figure 5A,C). Furthermore, protein analysis revealed that PSD-95 protein levels were markedly reduced in CRS mice, and BDT treatment reversed this decline (Figure 5B,D). Collectively, these findings demonstrate that BDT repairs CRS-induced synaptic structural and functional impairments by targeting core synaptic proteins such as PSD-95 and SYN1, providing direct experimental evidence for its role in enhancing synaptic plasticity.

### 2.6. Network Analysis

**Figure 6:**
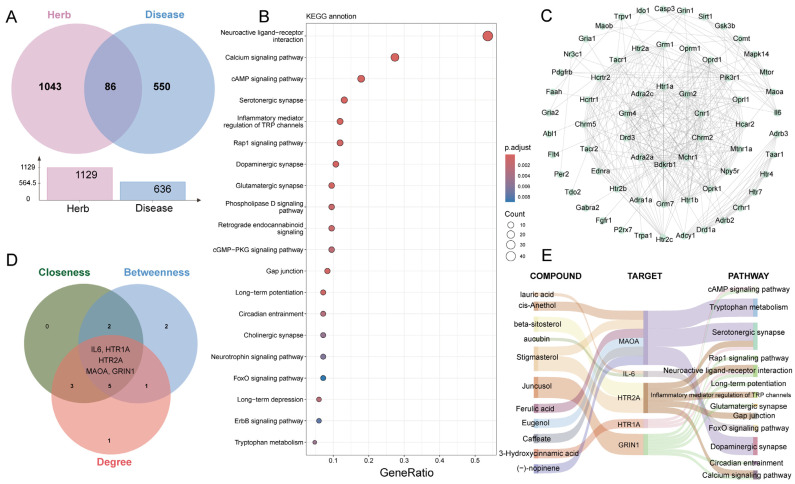
Network pharmacology analysis of BDT for depressive disorder treatment. (**A**) Venn diagram showing 1129 predicted targets of BDT components and 636 depressive-associated genes, with 86 overlapping genes identified as potential therapeutic targets. (**B**) Top 20 KEGG pathways enriched among the 86 overlapping genes, ranked by −lg(*p*-value). (**C**) PPI network of genes from the top 20 enriched pathways. (**D**) Hub gene identification using topological algorithms (BC/CC, Degree) via Cytoscape’s Hubba plugin. The intersection of top 10 genes from each metric revealed five core regulators: IL6, HTR1A, HTR2A, MAOA, GRIN1. (**E**) Systems pharmacology network integrating key BDT components (cis-anethole, stigmasterol, ferulic acid), their potential protein targets, and regulated pathways (serotonergic synapse, tryptophan metabolism). Edge colors denote interaction types: green (compound-target), blue (target-pathway).

To further validate that BDT alleviates depression by targeting MAOA and predict bioactive components modulating this mechanism, we employed network pharmacology analysis. Through comprehensive database mining and literature review, 1129 unique predicted targets of BDT components were identified, in which 636 genes were linked to depressive disorder. Intersection analysis identified 86 overlapping genes between BDT targets and disease genes (Figure 6A). Kyoto Encyclopedia of Genes and Genomes (KEGG) enrichment analysis of these 86 genes revealed the top 20 depressive-related pathways (Figure 6B). Protein–protein interaction (PPI) network analysis of pathway genes (Figure 6C), and subsequent topological screening using Cytoscape’s Hubba plugin (Cytoscape 3.7.2) (criteria: BC, CC, and Degree) prioritized 10 hub genes. Key candidates included IL-6, HTR1A, HTR2A, MAOA, and GRIN1 (Figure 6D). A “Compound-Target-Pathway” network (Figure 6E) was established, integrating major bioactive compounds (e.g., cis-Anethol, Stigmasterol, and Ferulic acid), targets, and biological pathways. Network analysis demonstrated that BDT components (e.g., Ferulic acid, Caffeate, Stigmasterol, (−)-nopinene, Eugenol, and cis-Anethol) directly interact with MAOA, modulating monoaminergic pathways such as serotonergic synapse and tryptophan metabolism, which collectively regulate 5-HT biosynthesis and synaptic signaling. Notably, the serotonergic synapse pathway regulates 5-HT receptor trafficking, while tryptophan metabolism controls 5-HT precursor availability. Based on these findings, we demonstrated that BDT alleviates depressive behaviors by enhancing 5-HT bioavailability through MAOA inhibition and potentiating downstream neuroplasticity via BDNF/TrkB activation.

## 3. Discussion

This study establishes that BDT mitigates depressive- and anxiety-like behaviors in a CRS-induced mouse model, as supported by behavioral, molecular, and neurochemical analyses. Based on these findings, we investigated the mechanisms by which BDT regulates serotonin metabolism and synaptic plasticity.

Central to this investigation is the role of MAOA, a key enzyme implicated in the degradation of monoaminergic neurotransmitters. MAOA, a flavoenzyme bound to the mitochondrial outer membrane, catalyzes the oxidative deamination of neurotransmitters and biogenic amines, including 5-HT, NE, and DA [18]. By degrading these monoamines, MAOA plays a pivotal role in the pathogenesis, progression, and treatment of neuropsychiatric disorders [11]. In depressive disorder, elevated MAOA expression and subsequent reductions in cerebral 5-HT and NE levels are recognized as key pathogenic factors. Preclinical studies have demonstrated that MAOA overexpression and its metabolic activity are closely linked to the development of neuropsychiatric diseases. For instance, in a chronic unpredictable stress (CUS)-induced rat model of depressive disorder, MAOA mRNA and protein levels were significantly increased in the telencephalon and hippocampus compared to controls, suggesting that MAOA accelerates monoamine metabolism, depletes neurotransmitter availability, and exacerbates depressive phenotypes [19]. Moreover, MAOA upregulation correlates strongly with depressive disorder, while MAO inhibitors effectively ameliorate depressive symptoms [20]. MAOA also interacts bidirectionally with elevated reactive oxygen species (ROS). ROS enhances MAOA activity, accelerating monoamine degradation, while MAO-mediated ROS overproduction further impairs mitochondrial function and reduces cellular viability [21,22]. Through network pharmacology, our study inferred ferulic acid, caffeate, stigmasterol, and eugenol in BDT as key compounds that inhibit MAOA activity, thereby improving serotonergic and dopaminergic synaptic transmission and enhancing tryptophan metabolism to promote 5-HT synthesis while suppressing its reuptake and degradation. Prior studies corroborate that ferulic acid [23] and eugenol [24], two bioactive constituents of BDT, exhibit potent MAOA inhibitory effects, increase 5-HT bioavailability, and demonstrate significant antidepressant potential.

5-HT, a pivotal neurotransmitter, plays critical roles in regulating mood, sleep, appetite, and other physiological functions. In depressive disorder, dysregulation of the 5-HT system is recognized as a key contributor to low mood and associated symptoms [25]. Studies indicate that 5-HT modulates neurotransmitter release and neuronal plasticity through interactions with specific receptor subtypes, such as HTR1A and HTR2A. Abnormal activation of HTR1A is associated with symptom alleviation, while increased HTR2A receptor density may correlate with disease progression [26,27,28]. Notably, synergistic effects between HTR2A antagonists and SSRIs have been shown to enhance antidepressant efficacy [29,30]. In our study, 3-hydroxycinnamic acid in BDT was found to regulate neuroactive ligand-receptor interactions, cAMP signaling pathway, and serotonergic synapse via HTR1A, influencing 5-HT secretion, receptor binding, and downstream biological processes. Additionally, stigmasterol and β-sitosterol in BDT act on HTR2A, modulating 5-HT secretion, ligand-receptor signaling (e.g., serotonergic synapse, gap junction, calcium signaling), and inflammatory pathways such as inflammatory mediator regulation of TRP channels. These findings suggest that BDT components exert antidepressant effects through multi-pathway mechanisms, providing a scientific foundation for developing novel therapies.

**Figure 7:**
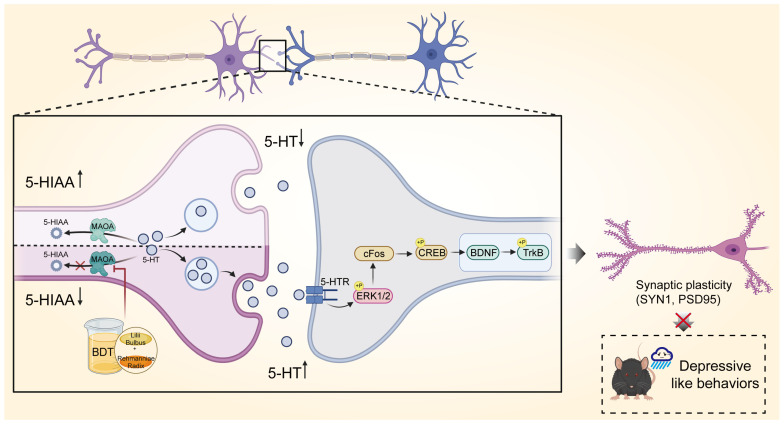
The mechanism of BDT in antidepressant effect. This integrated mechanism highlights the ability of BDT to synergistically regulate neurotransmitter metabolism, neurotrophic signaling, and synaptic plasticity, offering a novel therapeutic strategy for depressive disorder. (Arrows: ↑ upregulation, ↓ downregulation, red ×: Blockade.)

This study, through the construction of a C-T-P network, reveals the therapeutic mechanisms of BDT. Specifically, its active components synergistically regulate tryptophan metabolism and monoaminergic neurotransmission by targeting MAOA, while achieving cross-pathway modulation through integrated mechanisms. Mechanistically, MAOA inhibition reduces 5-HT degradation directly and elevates synaptic neurotransmitter concentrations (as reflected by the 5-HT/5-HIAA ratio), thereby activating downstream neuroprotective pathways. Crucially, BDT-mediated BDNF/TrkB signaling activation and synaptic marker upregulation (SYN1, PSD-95) form a cascading effect: elevated 5-HT levels enhance cAMP-PKA signaling via HTR1A receptors [31], promoting BDNF release and synaptic plasticity [32]. Improved synaptic plasticity, in turn, feedback-regulates monoamine homeostasis, establishing a “neurotransmitter metabolism-neurotrophic signaling-synaptic plasticity” positive feedback loop. Notably, beyond the classical monoamine hypothesis, network pharmacology predicted that BDT components (e.g., stigmasterol, β-sitosterol) might modulate 5-HT receptor subtypes (HTR1A and HTR2A). While experimental validation focused on MAOA and synaptic plasticity, these computational predictions suggest a broader regulatory role in serotonergic neurotransmission. For instance, the hypothesized differential modulation of HTR1A (activation) and HTR2A (antagonism) could balance 5-HT signaling dynamics, a mechanism analogous to the clinical strategy of combining SSRIs with HTR2A antagonists [29,30]. Collectively, this intervention strategy highlights BDT’s systemic regulation via C-T-P networks, offering novel insights to overcome conventional therapeutic limitations (Figure 7).

While this study advances our understanding of the antidepressant mechanisms of BDT, several limitations should be acknowledged. It is important to emphasize that our study, while revealing a strong correlation between MAOA inhibition and BDNF/TrkB pathway activation following BDT treatment, does not provide direct genetic or pharmacological evidence for a definitive causal relationship. Future studies are needed to determine whether MAOA downregulation is a necessary or sufficient condition for BDNF/TrkB activation. To unequivocally test the causal role of this axis, subsequent work could involve interventions such as hippocampal MAOA overexpression using viral vectors or co-administration of a selective TrkB antagonist (e.g., ANA-12) during BDT treatment. These approaches would directly assess whether disrupting the MAOA-BDNF/TrkB link abrogates the therapeutic effects of BDT. Additionally, network pharmacology suggests BDT may modulate 5-HT receptor sensitivity to influence 5-HT bioavailability, yet this hypothesis still requires experimental validation. As a multi-component herbal formula, BDT likely exerts its antidepressant effects via interactions with other neurotransmitter systems—such as dopamine and glutamate, which are critical in mood regulation and synaptic plasticity—but these were not explored in the current work. Future investigations should therefore examine the effects of BDT on dopaminergic and glutamatergic pathways, and further dissect the synergistic interactions among its bioactive constituents. Such efforts will help establish a comprehensive mechanistic framework to guide the optimization of BDT formulations and strengthen its clinical translation.

## 4. Materials and Methods

### 4.1. Preparation of BDT

*Lilium brownii* var. *viridulum* (fresh bulbs of *Lilium brownii*, Baihe in Chinese) and *Rehmannia glutinosa* (fresh roots of *Rehmannia glutinosa*, Dihuang in Chinese) were provided by China Shineway Pharmaceutical Group Limited (Shijiazhuang, China) and identified by Dr. Xiang of Hangzhou City University. The samples were stored in the School of Medicine, Hangzhou City University (Hangzhou, Zhejiang, China). According to the guidelines in the “Classic Prescription Key Information Table”, BDT was prepared using fresh bulbs of Baihe from Longshan, Hunan Province, China, and fresh roots of Dihuang from Jiaozuo, Henan Province, China. Firstly, 200 mL of Dihuang juice was extracted from the fresh roots; then, 245 g of Baihe was decocted with 400 mL of water until the volume was concentrated to 200 mL. Subsequently, the Dihuang juice was added to the *Baihe* decoction and further decocted until the volume was concentrated to 300 mL [33]. The 300 mL of BDT was solution was concentrated to 200 mL using rotary evaporation, and the final dosage for mice was determined to be 0.02 mL/g/day.

### 4.2. Quality Control of BDT

Precisely measure 4 mL of the Baihe Dihuang Tang decoction, dilute to 10 mL with purified water, and sonicate for 20 min. Centrifuge the sonicated aqueous solution at 12,000 rpm for 15 min. Collect the supernatant, filter through a 0.22 μm membrane, and prepare the aqueous sample for analysis by high-performance liquid chromatography (HPLC). The basic parameters of the HPLC system are listed in Table 1, and the gradient elution program is detailed in Table 2.

### 4.3. Animals and Model Construction

Male C57BL/6J mice (body weight: 20 ± 5 g) were obtained from GemPharmatech Co., Ltd. (Nanjing, China, SCXK (SU) 2023-0009). Mice were housed under controlled conditions (60 ± 5% relative humidity, 24 ± 2 °C) with a standardized 12 h light/dark cycle. After a 7-day acclimation period, mice were randomly assigned to three groups: control (CON), model (CRS), and BDT treatment (BDT). CON and CRS groups received equal volumes of saline. For except for CON group, CRS and BDT groups were subjected to CRS modeling. The CRS protocol was adapted from established methods [34,35,36]. Mice were placed in ventilated 50 mL conical tubes (multiple 2 mm air holes) with cotton padding at the posterior end to maintain restraint. Restraint was conducted daily from 08:00 a.m. to 10:00 a.m. for 2 h, with the modeling period lasting 14 consecutive days. During the modeling period, BDT group received daily intragastric administration of the decoction for 14 days (Figure 2A). All animal experiments and associated procedures received approval from the Animal Ethics Committee of Hangzhou City University (Approval No. 24040) on 25 June 2024.

### 4.4. Behavioral Testing

#### 4.4.1. Open Field Test (OFT)

An open-field box (60 cm × 60 cm × 40 cm) with a white flooring was subdivided into 16 grids, including 12 peripheral zones along the borders and 4 central grids. The box was constructed with four white opaque walls. Each mouse was gently placed at the central starting point and allowed to freely explore the box. After a 30 s habituation phase, the average speed and distance traveled within the margin areas were recorded over a 5 min observation period [37].

#### 4.4.2. Elevated Plus Maze (EPM)

The test apparatus is a plus-shaped maze elevated above the floor [37], consisting of four arms connected at the center by a square platform. Two opposing arms are “open”, lacking walls, while the other two opposing arms are “closed”, enclosed by walls that are often opaque to facilitate detailed behavioral observations. During the test, a mouse is placed in the central area and allowed to explore the maze freely for 5 min. The time spent in the open and closed arms, as well as the number of entries into each arm, is recorded for subsequent analysis.

#### 4.4.3. Forced Swimming Test (FST)

Mice were placed in a transparent cylindrical acrylic container (60 cm in height and 20 cm in diameter) filled with tap water maintained at a temperature of 22–24 °C and a water depth of 45 cm. They underwent a 6 min swimming acclimation period, during which the immobility time of the hind limbs was recorded during the final 4 min. The cumulative immobility time was used as an indicator to evaluate despair and depressive-like behavior in the mice [37].

#### 4.4.4. Tail Suspension Test (TST)

The test involved suspending a mouse by a rope attached 2 cm from the base of its tail, with the rope secured to a shelf, positioning the mouse’s head approximately 15 cm above the bottom of the testing apparatus. The mouse’s movements were video-recorded for 6 min, with data from the last 4 min used for analysis. Consistent with the FST, the cumulative immobility time was measured to assess despair and depressive-like behavior in the mice [37].

### 4.5. Brain Tissue and Blood Samples Processing

Animal sacrifice was performed via cervical dislocation. Brain tissues, including the hippocampus, were harvested under low-temperature conditions (on ice), placed in Eppendorf tubes, and stored at −80 °C until further use.

### 4.6. Examination of 5-HT and 5-HIAA

Approximately 10 mg of hippocampal tissue was mixed with 100 μL of PBS (pH 7.4) at 4 °C in a weight-to-volume ratio of 1 g:10 mL and homogenized thoroughly. The homogenate was then centrifuged at 3000× *g* for 20 min at 4 °C. The supernatant was collected, with one portion used for analysis and the remainder stored under cryopreservation. The concentrations of 5-HT and 5-HIAA in the hippocampus were quantified using commercially available ELISA kits of Nanjing Jiancheng Co., Ltd. (Nanjing, China) (Supplementary Table S1) following the manufacturer’s instructions, and the 5-HT/5-HIAA ratio was calculated.

### 4.7. RNA Extraction and Quantitative Real-Time PCR (qRT-PCR)

In this experiment, the total RNA extraction was performed using a kit with hippocampal tissue serving as the sample source. After extraction, the concentration of the isolated RNA was accurately measured using the NanoReady instrument of Hangzhou LifeReal Biotechnology Co., Ltd. (Hangzhou, China). Primers were designed and synthesized by Tsingke Biotechnology Co., Ltd. (Beijing, China), and detailed information on the primers for each target gene can be found in Supplementary Table S2. Subsequently, RNA was used to synthesize cDNA with a cDNA Synthesis Kit of Vazyme Biotech Co., Ltd. (Nanjing, China) (Supplementary Table S1). The qRT-PCR was conducted using Power SYBR Green PCR Master Mix of Vazyme Biotech Co., Ltd. (Nanjing, China) (Supplementary Table S1). Gene expression levels were normalized against β-actin using the 2^−ΔΔCt^ method [37].

### 4.8. Western Blotting

Thawed mouse hippocampal tissues were homogenized using an ultrasonic processor in RIPA solution containing phenylmethylsulfonyl fluoride (PMSF) protease inhibitors and phosphatase inhibitors. All samples were then incubated on ice for 30 min and centrifuged at 12,000× *g* for 15 min, after which the supernatant was collected. Protein concentrations were determined using an Enhanced BCA Protein Assay Kit (Hangzhou, China). Subsequently, equal amounts of protein mixtures with Loading Buffer were subjected to electrophoresis on a 10% SDS-PAGE gel, followed by transfer to a PVDF membrane (Darmstadt, Germany) (Supplementary Table S1). After blocking non-specific sites at room temperature, the membranes were incubated overnight at 4 °C with the following primary antibodies: β-actin, MAOA, BDNF, TrkB, p-TrkB, Synapsin I, and PSD95. On the second day, the membranes were washed three times with TBST and incubated with secondary antibodies for 1 h, followed by three washes with TBST (Supplementary Table S3 for antibody concentrations). The immunoblots were then developed using Clarity Western ECL Substrate of Shanghai Tanon Science & Technology Co., Ltd. (Shanghai, China) for signal detection. β-actin was used as an internal control, and the results of the immunoblots were quantified using Image J software (ij154-win-java8).

### 4.9. Network Pharmacology Analysis

The chemical constituents of Baihe and Dihuang were systematically retrieved from three primary sources: the Traditional Chinese Medicine Systems Pharmacology Database (TCMSP; https://tcmspw.com/tcmsp.php, accessed on 18 July 2024) and peer-reviewed literature. The canonical SMILES structures of identified compounds were annotated using PubChem (https://pubchem.ncbi.nlm.nih.gov/, accessed on 20 July 2024) to facilitate subsequent target prediction analyses. Putative targets of the identified compounds were predicted using three complementary tools: the Herbal Ingredients’ Targets (HIT2.0; http://www.badd-cao.net:2345/search, accessed on 18 July 2024), the Similarity Ensemble Approach (SEA; https://sea.bkslab.org/, accessed on 22 July 2024) (*p* < 0.05), and the Search Tool for Interactions of Chemicals (STITCH; http://stitch.embl.de/, accessed on 22 July 2024). Redundant targets were removed to generate a non-redundant target list. Genes associated with depressive disorder were compiled from four major resources: DrugBank (https://www.drugbank.ca/, accessed on 22 July 2024), the Online Mendelian Inheritance in Man database (OMIM, https://omim.org, accessed on 22 July 2024), the Therapeutic Target Database (TTD, http://bidd.nus.edu.sg/group/cjttd/, accessed on 22 July 2024), and curated literature. The search query focused on “*Major Depressive Disorder*” and its related pathways. The overlapping targets between the predicted compound targets and depressive-associated genes were identified, representing potential therapeutic targets of BDT for depressive disorder treatment.

The *ClusterProfiler R* package (R-4.3.1) was used to perform functional enrichment analysis of common genes using the KEGG database, applying a significance threshold of *p* ≤ 0.05. Relevant literature was reviewed to validate and refine the identification of depressive-related pathways [38], and genes enriched in the top 20 pathways were subsequently extracted. The gene set was subjected to protein–protein interaction (PPI) analysis using the STRING database (https://string-db.org/, accessed on 22 July 2024) with a confidence score cutoff of 0.7. Isolated nodes were excluded to generate a high-confidence PPI network. The Hubba plugin in Cytoscape 3.6.0 was applied to the PPI network to calculate Betweenness Centrality (BC), Closeness Centrality (CC), and Degree. The top 10 genes ranked by each centrality measure were intersected to identify hub genes [39]. Using the identified hub genes as queries, the corresponding compounds were retrieved. A “Compound-Target” network was constructed to visualize and summarize the multiscale biological mechanisms underlying antidepressant effects of BDT.

### 4.10. Statistical Analysis

Data were analyzed using IBM SPSS Statistics 25 (USA) and are expressed as Mean ± SD. Statistical analysis was performed with a one-way ANOVA, and a *p*-value of less than 0.05 was considered to indicate a statistically significant difference.

## 5. Conclusions

BDT alleviates depressive- and anxiety-like behaviors primarily by suppressing MAOA expression, reducing 5-HT oxidative degradation and elevating hippocampal 5-HT bioavailability. Increased 5-HT levels concurrently enhance neurotrophic signaling (upregulated BDNF/TrkB) and synaptic plasticity-related proteins (PSD-95/SYN1), facilitating synaptic recovery. Network pharmacology confirmed MAOA targeting and identified its modulatory bioactive components. These findings elucidate the mechanistic basis for BDT’s antidepressant efficacy. Future research should analyze synergistic interactions among BDT components (e.g., ferulic acid, stigmasterol) to optimize precision therapies for mood disorders.

## Figures and Tables

**Table 1:** Basic parameter settings of the HPLC system.

Parameter	Data
HPLC Model	Agilent 1290-Infinity II (Santa Clara, CA, USA)
Column	Waters CORTECS T3 (100 mm × 2.1 mm, 1.6 μm) (Milford, MA, USA)
Column Temperature	35 °C
Mobile Phase A:B	A: Water (0.1% formic acid, *v*/*v*) B: Acetonitrile
Flow Rate	0.3 mL/min

**Table 2:** Gradient elution program.

Time (min)	Mobile Phase A (%)	Mobile Phase B (%)
0–6	100	0
6–11	100–97	0–3
11–17	97	3
17–22	97–90	3–10
22–29	90–85	10–15
29–35	85	15
35–45	85–35	15–65
45–48	35–10	65–90
48.5	10–100	90–0
52	100	0

## Data Availability

The data presented in this study are uploaded during submission as a Supplementary File [Dataset name: Research Data.xlsx.] and are openly available for readers upon request.

## Supplementary Materials

The following supporting information can be downloaded at: https://www.mdpi.com/article/10.3390/ph18121786/s1, Table S1: Reagents and Kit catalogue; Table S2: PCR primer sequence; Table S3: Antibody catalogue.

## Abbreviations

BDT	Baihe Dihuang Tang
5-HT	Serotonin (5-Hydroxytryptamine)
5-HIAA	5-Hydroxyindoleacetic acid
TCM	Traditional Chinese medicine
SSRI	Serotonin reuptake inhibitors
NE	Norepinephrine
CON	Control Group
EPM	Elevated plus maze
TST	Tail suspension test
PPI	Protein–protein interaction
TrKB	Neurotrophic receptor tyrosine kinase 2
SYN1	Synapsin 1
HTR2A	5-hydroxytryptamine receptor 2A
MAOA	Monoamine oxidase A
CUS	Chronic unpredictable stress
SST	Somatostatin
CRS	Chronic restraint stress
DA	Dopamine
BC	Betweenness Centrality
CC	Closeness Centrality
OFT	Open field test
FST	Forced swimming test
KEGG	Kyoto Encyclopedia of Genes and Genomes
BDNF	Brain-derived neurotrophic factor
PSD95	Postsynaptic density protein 95
HTR1A	5-hydroxytryptamine receptor 1A

## References

[1] Santomauro D.F., Mantilla Herrera A.M., Shadid J., Zheng P., Ashbaugh C., Pigott D.M., Abbafati C., Adolph C., Amlag J.O., Aravkin A.Y. (2021). Global prevalence and burden of depressive and anxiety disorders in 204 countries and territories in 2020 due to the COVID-19 pandemic. Lancet.

[2] Yasenok V., Baumer A.M., Petrashenko V., Kaufmann M., Frei A., Rüegger S., Ballouz T., Loboda A., Smiianov V., Seifritz E. (2025). Mental health burden of persons living in Ukraine and Ukrainians displaced to Switzerland: The mental health assessment of the Ukrainian population (MAP) studies. BMJ Glob. Health.

[3] Gałecki P., Mossakowska-Wójcik J., Talarowska M. (2018). The anti-inflammatory mechanism of antidepressants—SSRIs, SNRIs. Prog. Neuro-Psychopharmacol. Biol. Psychiatry.

[4] Xue X., Pan J., Zhang H., Lu Y., Mao Q., Ma K. (2022). Baihe Dihuang (Lilium Henryi Baker and Rehmannia Glutinosa) decoction attenuates somatostatin interneurons deficits in prefrontal cortex of depression via miRNA-144-3p mediated GABA synthesis and release. J. Ethnopharmacol..

[5] Peng L., Zhang X.F., Guo D.Y., Zhai B.T., Liang Y.J., Chen Z.Z., Zou J.B., Shi Y.J. (2022). Evaluation of the Clinical Efficacy of the Classic Prescription “Baihe Dihuang Decoction” Based on Meta-Analysis. Evid. Based Complement. Altern. Med..

[6] Tong Y., Dong L., Shu H., Yang Y., Bai Y., Wen J. (2023). Preclinical evidence evaluation of Xiaoyao san in treating chronic unpredictable mild stress model of depression based on meta-analysis. Phytomedicine.

[7] Wang M., Bi Y., Zeng S., Liu Y., Shao M., Liu K., Deng Y., Wen G., Sun X., Zeng P. (2019). Modified Xiaoyao San ameliorates depressive-like behaviors by triggering autophagosome formation to alleviate neuronal apoptosis. Biomed. Pharmacother..

[8] Hu C., Yang H., Zhao H., Huang S., Liu H., Zhang S., Tang L. (2024). Antidepressant mechanism of Baihe Dihuang Decoction based on metabolomics and network pharmacology. China J. Chin. Mater. Medica.

[9] Cao L.-H., Wang Z.-Z., Zhao H., Tian S., He H.-J., Miao J.-X., Huang S.-n., Wang X.-Y., Song Y.-G., Kang L. (2025). The microglial state transition as a novel mechanism by which fresh Baihe Dihuang decoction prevents depression by regulating SIRT1/HMGB1 signaling. Phytomedicine.

[10] Tang L., Liu J., Yang H., Zhao H.Q., Hu C., Ma S.J., Qing Y.H., Yang L., Zhou R.R., Zhang S.H. (2024). Microbiome Metabolomic Analysis of the Anxiolytic Effect of Baihe Dihuang Decoction in a Rat Model of Chronic Restraint Stress. Drug Des. Dev. Ther..

[11] Naoi M., Maruyama W., Shamoto-Nagai M. (2018). Type A monoamine oxidase and serotonin are coordinately involved in depressive disorders: From neurotransmitter imbalance to impaired neurogenesis. J. Neural Transm..

[12] Spies M., Murgaš M., Vraka C., Philippe C., Gryglewski G., Nics L., Balber T., Baldinger-Melich P., Hartmann A.M., Rujescu D. (2023). Impact of genetic variants within serotonin turnover enzymes on human cerebral monoamine oxidase A in vivo. Transl. Psychiatry.

[13] Feng X., Liu Y., Liu B., Wang D., Liu L., Zhu L., Liu H., Zhang C., Yang W. (2023). Metabonomics Study on the Intervention of Baihe Dihuang Decoction in Depressed rats. Acta Chin. Med. Pharmacol..

[14] Zhao H., Tang L., Liu Y., Jiang J., Lyu R., LIiu J., Long H., Wang Y. (2023). Mechanism of Baihe Dihuang Decoction activating AMPA receptor to improve anxiety and depression-like behavior in chronic unpredictability stress mice. China J. Tradit. Chin. Med. Pharm..

[15] Mao Q., Zhang H., Zhang Z., Lu Y., Pan J., Guo D., Huang L., Tian H., Ma K. (2024). Co-decoction of Lilii bulbus and Radix Rehmannia Recens and its key bioactive ingredient verbascoside inhibit neuroinflammation and intestinal permeability associated with chronic stress-induced depression via the gut microbiota-brain axis. Phytomedicine.

[16] Liu F., Jia Y., Zhao L., Xiao L.-n., Cheng X., Xiao Y., Zhang Y., Zhang Y., Yu H., Deng Q.-e. (2024). Escin ameliorates CUMS-induced depressive-like behavior via BDNF/TrkB/CREB and TLR4/MyD88/NF-κB signaling pathways in rats. Eur. J. Pharmacol..

[17] Zou J., Chen K., Zhang Z. (2024). Effects of the 5-HT1A receptor antagonist on synaptic plasticity in sevoflurane-induced cognitive dysfunction in aged rats and its mechanism. J. China Med. Univ..

[18] Ma Y., Chen H., Li H., Zhao Z., An Q., Shi C. (2024). Targeting monoamine oxidase A: A strategy for inhibiting tumor growth with both immune checkpoint inhibitors and immune modulators. Cancer Immunol. Immunother..

[19] Cui T., Xie W., Fu X.Y., Li H.Y., Jiang X.H., Qiu M.-H. (2016). Experimental study on abnormal expression of TPH2,DDC and MAO-A involved in depression-like behaviors of rats induced by CUS. Chin. Pharmacol. Bull..

[20] Suchting R., Tirumalaraju V., Gareeb R., Bockmann T., de Dios C., Aickareth J., Pinjari O., Soares J.C., Cowen P.J., Selvaraj S. (2021). Revisiting monoamine oxidase inhibitors for the treatment of depressive disorders: A systematic review and network meta-analysis. J. Affect. Disord..

[21] de Morais H., de Souza C.P., da Silva L.M., Ferreira D.M., Werner M.F., Andreatini R., da Cunha J.M., Zanoveli J.M. (2014). Increased oxidative stress in prefrontal cortex and hippocampus is related to depressive-like behavior in streptozotocin-diabetic rats. Behav. Brain Res..

[22] Rawdin B.J., Mellon S.H., Dhabhar F.S., Epel E.S., Puterman E., Su Y., Burke H.M., Reus V.I., Rosser R., Hamilton S.P. (2013). Dysregulated relationship of inflammation and oxidative stress in major depression. Brain Behav. Immun..

[23] Grzelczyk J., Budryn G., Peña-García J., Szwajgier D., Gałązka-Czarnecka I., Oracz J., Pérez-Sánchez H. (2021). Evaluation of the inhibition of monoamine oxidase A by bioactive coffee compounds protecting serotonin degradation. Food Chem..

[24] Tao G., Irie Y., Li D.-J., Keung W.M. (2005). Eugenol and its structural analogs inhibit monoamine oxidase A and exhibit antidepressant-like activity. Bioorg. Med. Chem..

[25] Żmudzka E., Sałaciak K., Sapa J., Pytka K. (2018). Serotonin receptors in depression and anxiety: Insights from animal studies. Life Sci..

[26] Cui L., Li S., Wang S., Wu X., Liu Y., Yu W., Wang Y., Tang Y., Xia M., Li B. (2024). Major depressive disorder: Hypothesis, mechanism, prevention and treatment. Signal Transduct. Target. Ther..

[27] Wu X., Yao J., Ding M., Shi Z.-S., Xu F.-L., Zhang J.-J., Wang B.-J. (2017). 5-HT1A receptor (HTR1A) 5′ region haplotypes significantly affect protein expression in vitro. Neurosci. Lett..

[28] Yohn C.N., Gergues M.M., Samuels B.A. (2017). The role of 5-HT receptors in depression. Mol. Brain.

[29] Hervig M.E.-S., Zühlsdorff K., Olesen S.F., Phillips B., Božič T., Dalley J.W., Cardinal R.N., Alsiö J., Robbins T.W. (2024). 5-HT 2A and 5-HT 2C receptor antagonism differentially modulate reinforcement learning and cognitive flexibility: Behavioural and computational evidence. Psychopharmacology.

[30] Kimura K.T., Asada H., Inoue A., Kadji F.M.N., Im D., Mori C., Arakawa T., Hirata K., Nomura Y., Nomura N. (2019). Structures of the 5-HT2A receptor in complex with the antipsychotics risperidone and zotepine. Nat. Struct. Mol. Biol..

[31] Parenti I., Leitão E., Kuechler A., Villard L., Goizet C., Courdier C., Bayat A., Rossi A., Julia S., Bruel A.-L. (2022). The different clinical facets of SYN1-related neurodevelopmental disorders. Front. Cell Dev. Biol..

[32] Gu Y., Pope A., Smith C., Carmona C., Johnstone A., Shi L., Chen X., Santos S., Bacon-Brenes C.C., Shoff T. (2024). BDNF and TRiC-inspired reagent rescue cortical synaptic deficits in a mouse model of Huntington’s disease. Neurobiol. Dis..

[33] Liu Y., Liu J., Li D., Sun B., Yang J., Fu Y., Ma S., Zhu G. (2024). Research Progress and Quality Marker Prediction of Famous Classical Formula Baihe Dihuangtang. Chin. J. Exp. Tradit. Med. Formulae.

[34] He Y., Ren Y., Chen X., Wang Y., Yu H., Cai J., Wang P., Ren Y., Xie P. (2024). Neural and molecular investigation into the paraventricular thalamus for chronic restraint stress induced depressive-like behaviors. J. Adv. Res..

[35] Li Y., Jia Y., Wang D., Zhuang X., Li Y., Guo C., Chu H., Zhu F., Wang J., Wang X. (2021). Programmed cell death 4 as an endogenous suppressor of BDNF translation is involved in stress-induced depression. Mol. Psychiatry.

[36] Wang Z., Jin S., Xia T., Liu Y., Zhou Y., Liu X., Pan R., Liao Y., Yan M., Chang Q. (2022). Nelumbinis Stamen Ameliorates Chronic Restraint Stress-Induced Muscle Dysfunction and Fatigue in Mice by Decreasing Serum Corticosterone Levels and Activating Sestrin2. J. Agric. Food Chem..

[37] Han H., Xu M., Wang J., Li M.D., Yang Z. (2024). CRISPR/Cas9 based gene editing of Frizzled class receptor 6 (FZD6) reveals its role in depressive symptoms through disrupting Wnt/β-catenin signaling pathway. J. Adv. Res..

[38] Yu G., Wang L.-G., Han Y., He Q.-Y. (2012). clusterProfiler: An R Package for Comparing Biological Themes Among Gene Clusters. OMICS A J. Integr. Biol..

[39] Sasikumar D.S.N., Thiruselvam P., Sundararajan V., Ravindran R., Gunasekaran S., Madathil D., Kaliamurthi S., Peslherbe G.H., Selvaraj G., Sudhakaran S.L. (2024). Insights into dietary phytochemicals targeting Parkinson’s disease key genes and pathways: A network pharmacology approach. Comput. Biol. Med..

