# Supplementary material for: Baihe Dihuang Tang Exerts Antidepressant Effects via Modulation of MAOA-Mediated Serotonin Metabolism and Synaptic Plasticity
Source: Pharmaceuticals (Basel). 2025 Nov 24;18(12):1786. doi: 10.3390/ph18121786 (PMC12736097; doi:10.3390/ph18121786)
Supplement: Supplementary file 1 [file pharmaceuticals-18-01786-s001.zip › Supplementary tables.pdf]

Supplementary Table S1. Reagents and Kit catalogue

Name	Catalog No.	Source
5-HT Kit	H104-1-2	Nanjing Jiancheng Co., Ltd
5-HIAA Kit	H411-1-2	Nanjing Jiancheng Co., Ltd
RNA Isolation Kit V2	RC112	Vazyme Biotech Co., Ltd
RT SuperMix Perfect for qPCR	R333	Vazyme Biotech Co., Ltd
Power SYBR Green PCR Master Mix	Q711	Vazyme Biotech Co., Ltd
RIPA	FD009	Fdbio science
PMSF	FD0100	Fdbio science
phosphatase inhibitors	FD1002	Fdbio science
BCA Kit	FD2001/2002	Fdbio science
SDS-PAGE gel	FD341	Fdbio science
Loading Buffer	FD002	Fdbio science
PVDF membrane	ISEQ00010	Merck Millipore Ltd.

Supplementary Table S2. PCR primer sequence

Gene Name	Primer Sequence (5′ to 3′)
MAOA	F: GCCCAGTATCACAGGCCAC<br>R: GTCCCACATAAGCTCCACCA
BDNF	F: GATCCACTGAGCAAAGCCGA<br>R: CACCTGGTGGAACATTGTGG
TrKB	F: CGGGAGGCCGGGGAA<br>R: ACCTCAGGGCTGGGGAG
PSD95	F: GAGATGTCCCAGAGACCAAGAG<br>R: ATAATAGTCCAGGATGTCCAGCAA
SYN1	F: GGATGTGCCACCACCCATCA<br>R: TGGTCAGAGACTGGGATTTGTTGA
β-actin	F: GCCTCCCTTCTTGGGTATGGA<br>R: AATGCCTGGGTACATGGTGG

Supplementary Table S3. Antibody catalogue

Name	Catalog No.	Source	Application/Dilution
MAOA	PB9664	Boster Biological Technology Co., Ltd.	WB/1:5000
BDNF	PAA011Mu01	CLOUD-CLONE CORP	WB/1:1000
TrKB	13129-1-AP	Proteintech Group, Inc.	WB/1:5000
p-TrKB	AF1963	Beyotime Biotechnology	WB/1:5000
PSD95	20665-1-AP	Proteintech Group, Inc.	WB/1:5000
SYN1	20258-1-AP	Proteintech Group, Inc.	WB/1:5000
β-actin	GB15003-100	Servicebio Technology Co., Ltd.	WB/1:5000
HRP (Goat Anti-Rabbit)	BA1054	Boster Biological Technology Co., Ltd.	WB/1:10000
